# The impact of climate change, population growth and development on sustainable water security in Bangladesh to 2100

**DOI:** 10.1038/s41598-022-26807-6

**Published:** 2022-12-26

**Authors:** Mac Kirby, Mohammed Mainuddin

**Affiliations:** CSIRO Land and Water, GPO Box 1700, Canberra, ACT 2610 Australia

**Keywords:** Climate sciences, Environmental sciences, Environmental social sciences, Hydrology

## Abstract

There are concerns that groundwater use for irrigation and for urban water supply is unsustainable in some parts of Bangladesh, particularly in the agriculturally productive northwest region. We use an integrated population – GDP – food – water model to examine water demand to 2100 in Bangladesh in development scenarios relevant to food and water security. The results indicate that irrigation water demand is projected to increase in coming decades, but later in the century it may decrease due to increasing crop yields and a falling population. The increased demand is greatest in the northwest region and, if unchecked, would increase concerns there about the unsustainable use of groundwater. The growth in demand is determined particularly by growth in crop yields, population growth and the fraction of food demand satisfied by imports. An extreme hot-dry climate change scenario has a lesser impact. This suggests that, in principle, Bangladesh can offset the impacts of climate change on irrigation water demand through its domestic policies. Urban water use currently also leads to concerns over unsustainable groundwater use. Our results suggest that urban water demand is likely to grow proportionately significantly more than irrigation water demand. Alternative sources for urban water are therefore urgently required.

## Introduction

Many developing nations face challenges with increasing population, growing demand for food and hence irrigation water, and unsustainable use of water. Climate change may exacerbate the challenges^1^. Developing nations also aim to increase economic development to tackle poverty, which will require an even greater use of resources, including water^2^. Groundwater use globally is particularly important, and its use may involve trade-offs amongst sustainable development goals, such as protecting groundwater on the one hand, and food security on the other^3^.

At the national level, Bangladesh does not experience water scarcity, but does suffer from low water security due to extreme floods, poor water quality and transboundary water issues^4^. However, the national picture obscures local and seasonal water scarcity, which has led to the unsustainable use of groundwater for irrigation in parts of the northwest region^5–13^. Climate change may exacerbate the problems with sustainable use in the northwest^14^, as will population increase and the increase in demand for food^15^.

Other factors that will influence future irrigation water demand include the choice of crops, particularly a reduction in the future requirement for rice, a high-water using crop^16^. With the achievement of near self-sufficiency in rice, it may be preferable in future to limit the growth of rice production^17^, and instead grow crops which use less water, such as pulses and oilseeds^18^. However, grain self-sufficiency has declined in recent years^19^. Changing diets to consume less animal protein may also reduce the demand for water^20^, though in the case of Bangladesh (and many other countries) supplying the whole population with a recommended diet would increase water consumption^21^. Increases in crop yields will also affect future water demand^15^, as will the impact on local crop production of the quantity and type of food imports and exports.

Urban water supply in Bangladesh’s biggest city, Dhaka, is also based on the unsustainable use of groundwater, with rapid depletion threatening both the quantity^22–24^ and quality^25^ of the resource. Population increase will again exacerbate the problems^26^. Urban water demand will also be affected by the rate of rural to urban migration.

In response to the problems and opportunities noted above, the Government of Bangladesh has developed a range of policy responses^27–30^. These plans include measures to sustain and increase overall agricultural production of the country due to rapid conversion of agricultural area for urban and industrial use in the central region^31,32^, to reduce Boro rice cultivation in the northwest region for sustainability of groundwater irrigation, to enhance the productivity of Transplanted Aus rice and dry season irrigated Boro rice to increase food security of the country, to expand surface irrigation in the southwest and northeast region, effectively addressing the overwhelming dependence on groundwater, and focusing on increasing the use of surface water. Similarly, the Dhaka Water Supply and Sewerage Authority aims to reduce the dependency on groundwater by increasing supply from the surface water sources^33^. Despite all these long-term and short-term plans, there are no comprehensive water security scenarios for the future considering the economic development, population growth, and climate change.

**Figure 1:**
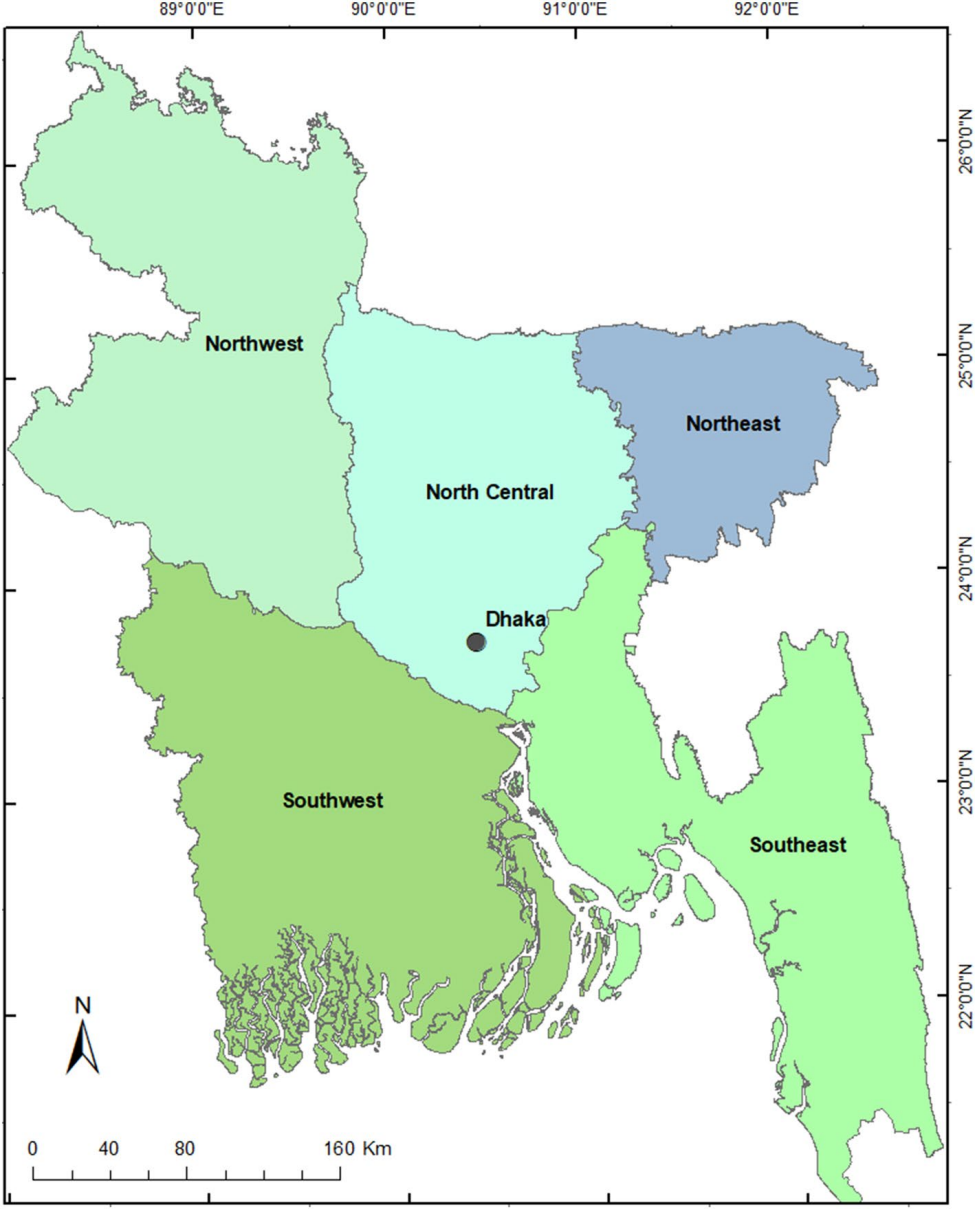
Outline map of Bangladesh with the five regions examined in this paper. The Northwest region comprises the Rangpur and Rajshahi divisions of Bangaldesh; North Central, the Mymensingh division and Dhaka division north of the Padma River; Northeast, Sylhet division; Southwest, Khulna and Barisal divisions and Dhaka division south of the Padma River; Southeast: Chittagong division.

Our aim here is to examine development trajectories of irrigation and urban water demand to 2100 for Bangladesh under a range of assumptions about future development and climate change. We examine the demand trajectories for five regions of Bangladesh (shown in Fig. 1), enabling us to examine whether the demand is likely to place further pressure on the northwest region in particular, leading to more unsustainable use of groundwater, or whether the demand can be mitigated, easing pressure on groundwater in that region. We use the model of Kirby and Mainuddin^34^, which includes the effects of: population growth; economic growth; the import and export of main food types; agricultural yield increases; changed regional cropping patterns; the impacts of climate change on rainfall and crop water demand; and the impacts of population growth and rural–urban migration on urban water demand. Some aspects of the model are similar to an earlier model for Pakistan^35^, but that model and the current model differ in several important ways (described later in the Methods section). In the model, population growth and economic growth are linked, and co-evolve in a way which matches the observed development in Bangladesh from 1975 to 2020, and the expected development in the future, based on a similar population – GDP model for Pakistan^36^. No study is known to us that examines this range of potential impacts on future irrigation and urban water demand.

The novel contribution in this paper is the range of effects considered under a single integrated analysis, in particular the integration of crop water demand (including climate change impacts) and urban water demand with socio-economic effects of population growth and GDP growth. We also consider projections to 2100, which is further ahead than most studies (e.g. Mainuddin and Kirby^15^, who assessed projections to 2050) and also matches with the Basin Development Plan 2100. While the model used here considers socio-economic effects, it is strictly not an economics model with supply – demand – price effects. We will comment on this in the methods section below and in the discussion section.

## Methods

### Integrated model of population growth, GDP growth, food supply and water security

**Figure 2:**
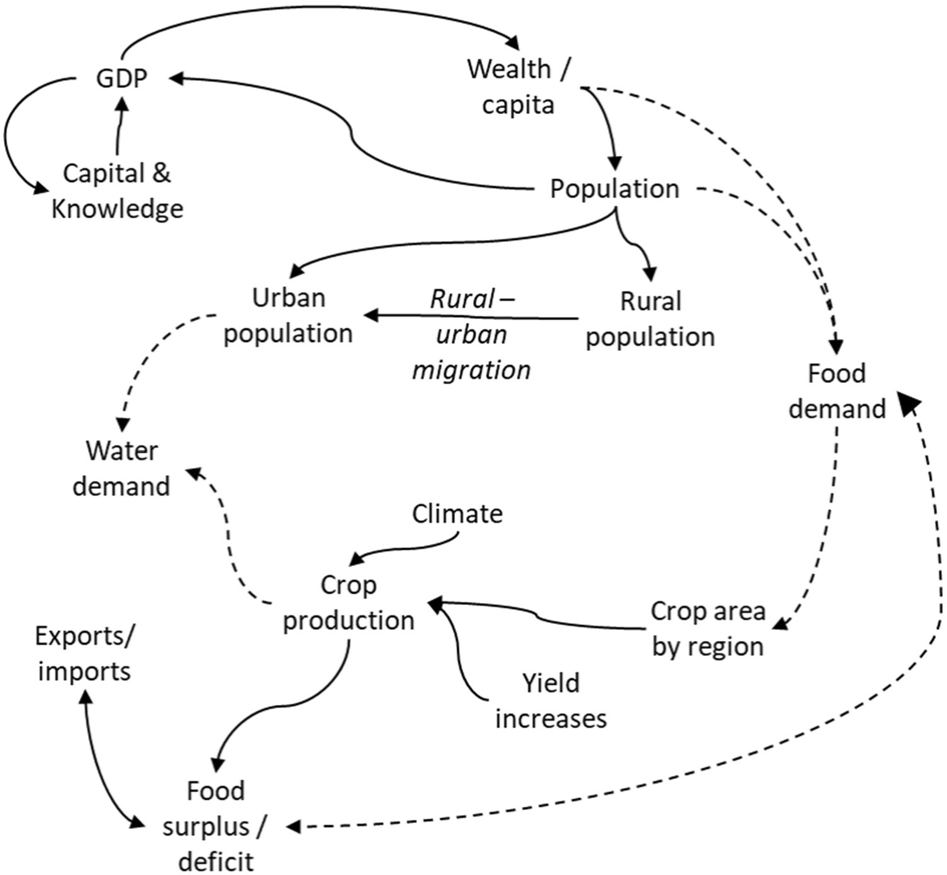
Schematic diagram of the population – GDP – food – water model. The names represent a quantity that is simulated by a sub-model or, in the case of climate, an input sourced from an external model. The lines indicate links amongst sub-models, with dashed lines indicating links that represent a demand for food or water.

The model is fully described in Kirby and Mainuddin^34^. Briefly, the model incorporates all the effects shown schematically in Fig. 2. It is based on blending a long-run development model of population and GDP growth^37,38^, with a physically based food demand model similar to but simpler than that of Bijl et al.^39^ and a crop coefficient water demand model^40^, together with other sub-models to simulate food importation and rural to urban migration. Figure 2 gives a schematic picture of the overall model. Population growth is mediated by economic development (in particular, the population growth rate slows with increasing per capita wealth). The population demands food (with the dietary mix changing as per capita wealth increases), which in turn requires a mix of crops to be grown. The crops in turn demand water, some of which is supplied by rain, but some is supplied by irrigation water particularly in the dry season. As the economy and population grow, food demand grows, and hence the area of crops and volume of irrigation water required to satisfy the food demand also grow, though the growth is offset by increasing crop yields. Some food is supplied by imports, and some may also be exported. The domestic food production is shared amongst five regions of Bangladesh (Fig. 1); this sub-national modelling is done to examine in more detail the pressures on the northwest region where the water security concerns are greatest, and also to examine the potential impact of development elsewhere to ease those pressures. Urban water demand is also examined; population growth plus rural-to-urban migration lead to urban population growth, and hence growing urban water demand.

Water supply in Bangladesh is not physically constraining: the flow of all the rivers entering Bangladesh is considerably greater than the demand for water even in the dry season (though there are competing requirements for using that water, such as for the environment, salt flushing in the delta, and the maintenance of depths for river transport vessels). Therefore, the model does not consider water supply. Based on the interactions with population, etc., described above, the model assesses the demand for water nationally, as well as regionally. While it may be preferable in many cases to study water use and demand within hydrological units such as a river basin, here we deal with demand only as it is managed nationally and regionally, hence our use of the regions shown in Fig. 1. We use the demand as an indicator of likely future sustainability. The model has some common features with the earlier model of Pakistan due to Kirby and Ahmad^35^, but differs in many respects. In particular, the Pakistan model considers water supply, whereas the current Bangladesh model does not, as explained above. In contrast to the Pakistan model, the current model assesses urban demand and we also use it to assess regional demand whereas the Pakistan model was used only at the national level^35^.

The model can simulate reasonably well the historical (1975 to present) evolution of a wide range of features of Bangladesh^34^ including: population growth (with urban and rural population split); GDP growth; food availability, export and import; the areas, yield and production of seven main crop types in five regions of Bangladesh; crop water demand in the five regions; and urban water demand both overall and in Dhaka, the largest urban centre. The model also simulates future trends that compare with other projections^34^.

The model outlined above does consider socio-economic effects, but is not an economics model with supply – demand – price effects. In particular, the imports and exports are not modelled as trade with an external (world) market; they are modelled simply to be deposited into, or withdrawn from an external pool. The effects of external trends and shocks such as the global food crisis of 2008 or the current crisis in Ukraine are large and largely unpredictable, and the response is as much political as it is purely economic. We think, therefore, that it is safer to model the consequences for the physical demand for water of effects resulting from changes to exports and imports, rather than attempting to model the economics of and responses to external trends and shocks.

The data inputs for the model to simulate a base case (business as usual) scenario are described in detail by Kirby and Mainuddin^34^ who give the sources of data and values for all parameters covering a wide range of inputs for GDP and population growth, food demand per capita, food imports, crop area and cropping intensity by region, crop water demand, and urban water demand, together with time series of rainfall and reference evapotranspiration. Many of the data, such as GDP and population, are at the national level, whereas data covering crop areas and yield are based on district level (i.e. at a finer spatial level than the regions of Fig. 1) data from the Bangladesh Bureau of Statistics.

### Scenarios

We assessed projections under a base case (business as usual) and with changes which might result from socio-economic effects (changes to population growth rates and, GDP growth rates), agricultural and water management (crop yield increase, changing the crop mix with changed dietary preferences, reducing crop areas in the northwest), and climate change. We also assessed the impact on urban water demand of changes to population growth rates, GDP growth rates, and rural–urban migration rates. Some of these scenarios are similar to the earlier study in Pakistan^35^, but several are specific to the Bangladesh case: these include scenarios of rural to urban migration and urban water demand, food importation, and changed regional cropping patterns.

#### Base case

The base case is a simulation of the development of population, GDP, food supply, and water demand in the absence of climate change or other changes. The climate for the base case is the historical climate time-series (of precipitation, potential evapotranspiration and average temperature) from 1975 to 2020, repeated from 2021 onwards. The base case is identical to the example given in Kirby and Mainuddin^34^.

#### Changed rate of population growth

The rate of population growth in the base case is that which simulates the population growth to 2100 of the median projection of the UN^41^. The rate of population growth from 2020 onwards was changed in two scenarios, one of which resulted in a 14% larger population in 2100, and the other of which resulted in a 24% lower population. These scenarios are well within the UN Population Division’s probabilistic + /− 80% scenarios of a 32% smaller or a 35% larger population in 2100^42^.

#### Changed rate of rural to urban migration

The rate of urban population growth depends partly on the general rate of population growth and partly on rural to urban migration. In the base case, the urban population grows to 121 m by 2100. The rate of rural to urban migration was varied in two scenarios, in the first of which the urban population grows to 139 m and in the second it grows to 104 m.

#### Changed rate of GDP growth

The rate of GDP growth from 2020 onwards was changed in two scenarios, such that annual GDP growth is 2% larger or 2% smaller. The first resulted in a 7% larger population in 2100, and the other in a 6% smaller population in 2100. The GDP change results in a population change, but because it also impacts factors such as food demand (due to a wealthier population demanding more food), these scenarios are not direct substitutes for population change scenarios.

#### Changed rate of food importation growth

Rice imports have fallen as a proportion of overall supply in the last few decades, although they have grown in absolute quantity; imports of some other food including wheat have grown both absolutely and as a proportion of supply^43^. The rate of change of imports as a fraction of overall supply was changed in two scenarios, in the first of which the rate of imports grows faster to 2100, and in the second of which it grows slower with the import of some foods (particularly rice, and pulses and oilcrops) ending in the later part of the century.

#### Changed dietary preferences

Dietary preferences were changed in two scenarios. In the first, the amount of maize per capita (produced primarily as feed for meat production^19^) was increased progressively from the current value to ten times that in 2100. In the second scenario, the amount of wheat per capita was increased progressively from the current value of about 17 kg/cap/year to three times that in 2100; the amount of rice was decreased by the same amount. The reduction in rice consumption was assumed to all come from a reduction in the *Boro* crop, because of the overlap of the wheat and *Boro* cultivation seasons.

#### Changed yield increases

In the base case, agricultural yields are projected to grow at the same rate as they did historically until yields are 1.5 times the current yields. Once this increase is attained, there is no further yield increase. The multiple of current yields at which further increase stops was set at 1.1 in one scenario and at 2 in a second.

#### Focus on development of agriculture in the southwest, and decrease in the northwest

The greatest growth in crop area and hence greatest pressure on groundwater resources is in the northwest of Bangladesh, as noted in the introduction. In one scenario, the rate of increase in future crop area in the southwest from 2021 to 2100 was increased to match that of the historical rate of increase in the northwest, and the future rate of increase in the northwest was correspondingly reduced.

#### Climate change

Climate change projections of the 6th Assessment Report of The Intergovernmental Panel on Climate Change can be obtained for individual countries or river basins from the IPCC WG1 Interactive Atlas at https://interactive-atlas.ipcc.ch/. The data available from the IPCC includes change factors for 2030, 2050, and 2100. We used the P10 and P90 (10 percent and 90 percent) rainfall and temperature projections from the most extreme scenario SSP5-8.5. We assessed the change in potential evapotranspiration from the change in maximum and minimum temperatures using the Hargreaves Equation.^44^. We combined the driest and hottest (greatest potential evapotranspiration) to form an extreme hot-dry scenario, and the wettest and least hot (least potential evapotranspiration) to form a warm-wet scenario. The changed climate values in the scenarios are summarised in Table 1. We assumed that the changes varied linearly from 2021 to 2030, from 2030 to 2050, and from 2050 to 2100.

**Table 1:** Two projected climate change scenarios, with the changed climate values in 2030, 2050 and 2100.

Scenario	Year	Precipitation change	Potential evapotranspiration change
Hot-dry	2030	0.94	0.99
Hot-dry	2050	0.96	1.01
Hot-dry	2100	1.09	1.14
Warm-wet	2030	1.13	0.98
Warm-wet	2050	1.20	0.99
Warm-wet	2100	1.52	1.04

## Results

The integrated model results in the output of many variables, each as a time series from 1960–2100. These include population, GDP, food demand, food imports, areas of crops (for each of seven crop groups in five regions), and demand for water^34^. Here we focus on the time-series of irrigation and urban water demand as indicators of water security, and on the population and GDP per capita (as an index) in 2100. We examine the total volume of demand projected in each of the five regions.

### Base case

**Figure 3:**
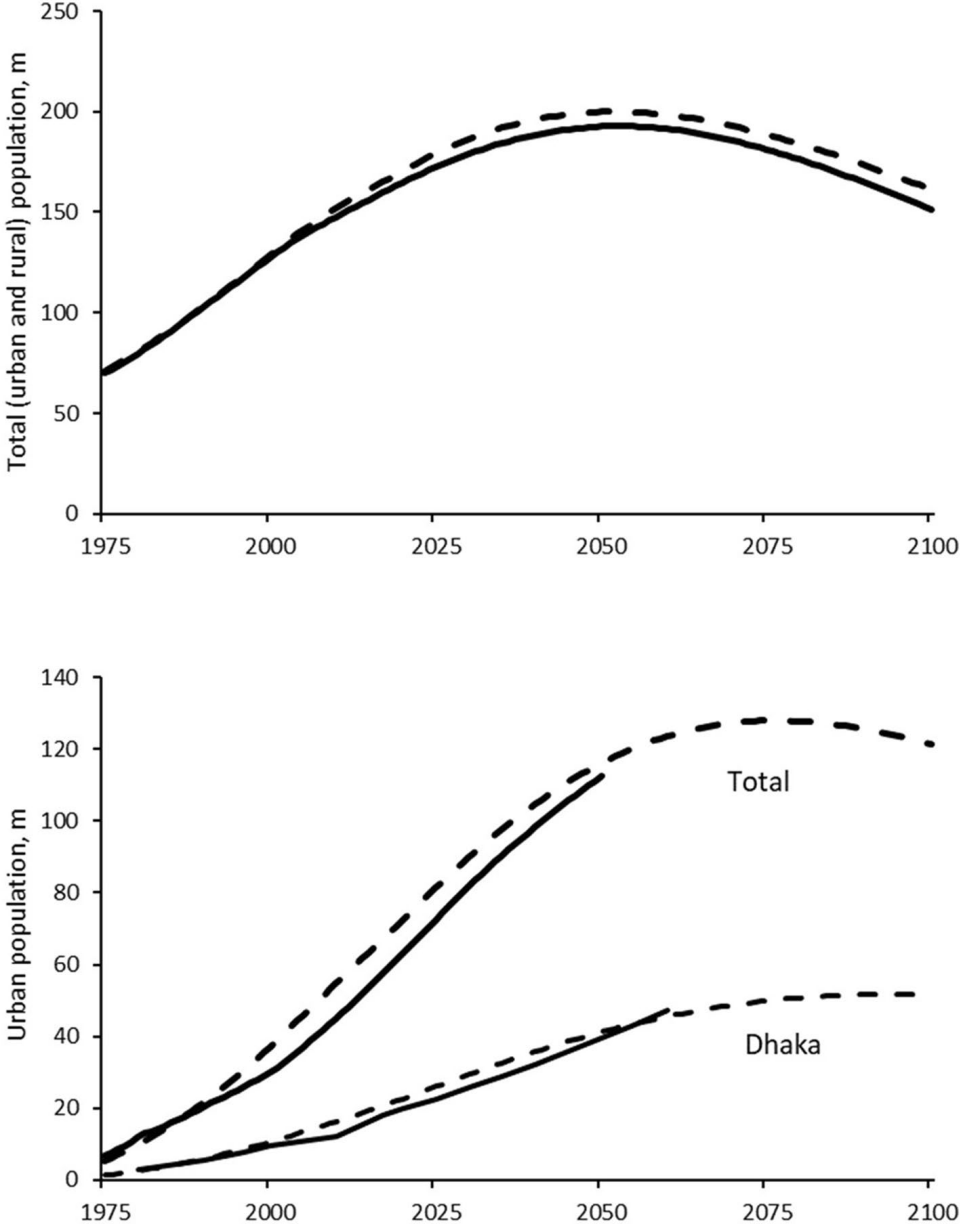
Total country population (top) and urban population (bottom), with the latter showing the total urban population and that of Dhaka, the largest urban centre. The solid lines are from the UN^41^ (total population, top), UN^45^ (total urban population (bottom) and the Dhaka population according to Rahman and Qauyyum^46^ (1975 – 2020) and Haque^26^ (2020 – 2060). The dashed lines show model output.

The total and urban population from 1975 to 2100 in the base case is shown in Fig. 3. The simulated population growth is similar to the median projection of the UN^41^. The GDP (as an index, with 1975 = 100) and GDP per capita (GDP as an index divided by population) in the base case are shown in Table 2. The GDP and GDP per capita in 2100 are approximately 50 times that in 2020.

**Table 2:** Population, GDP (as an index, with 1975 = 100, GDPIx) and GDP per capita (GDP as an index divided by population, GDPIx/cap) in the base case and scenarios.

Scenario	Population, m				GDPIx	GDPix/cap
	Total	Urban	Rural	Max		
Base	161	121	40	200	44,227	274
High population growth	184	138	45	213	45,279	247
Low population growth	122	92	30	183	42,055	345
High GDP growth	152	114	38	198	99,036	651
Low GDP growth	173	130	43	202	19,579	113
High rural to urban migration	161	139	22	200	44,227	274
Low rural to urban migration	161	104	57	200	44,227	274

The total, urban and rural population in 2100 is shown, together with the maximum population reached between the present and 2100. Note that in the case of scenarios not listed, the population, GDP and GDP per capita are the same as in the base case.

**Figure 4:**
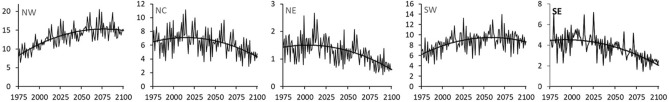
Annual volume demand in billion cubic metres for irrigation water in the base case for the five regions of Bangladesh. The thicker smooth lines are fitted functions of the form *Y* = *a1* + *a2.X* + *a3.x*^2^. Note that the scales differ in the different plots.

The water demand for irrigation in the base case (i.e. business as usual water management and use) is shown in terms of total volume of demand in Fig. 4. A key feature of the simulation is that the total volume demand for irrigation water is projected to peak in all regions and then decline, starting from about the present in the north central (NC), northeast (NE) and southeast (SE), from about 2060 in the southwest (SW) and from about 2080 in the northwest (NW) (Fig. 4). However, year-to-year variation in water demand will mask any decreases (or increases) for some years.

### Population and GDP in the scenarios

The population, GDP (as an index, with 1975 = 100) and GDP per capita (GDP as an index divided by population) in the scenarios at 2100 are shown in Table 2. Note that scenarios which involve cropping changes and climate change have the same population and GDP as the base case; the table shows only those scenarios in which population and/or GDP changes. The lowest population simulated is 122 m and the highest is 184 m. The GDP and GDP per capita in 2100 vary from about 23 times that of 2020 (in the low GDP case) to somewhat above 100 times that of 2020 (in the high GDP case). The urban population varies from 92 to 139 m, whereas it is 121 m in the base case.

### Irrigation water demand in the scenarios

**Figure 5:**
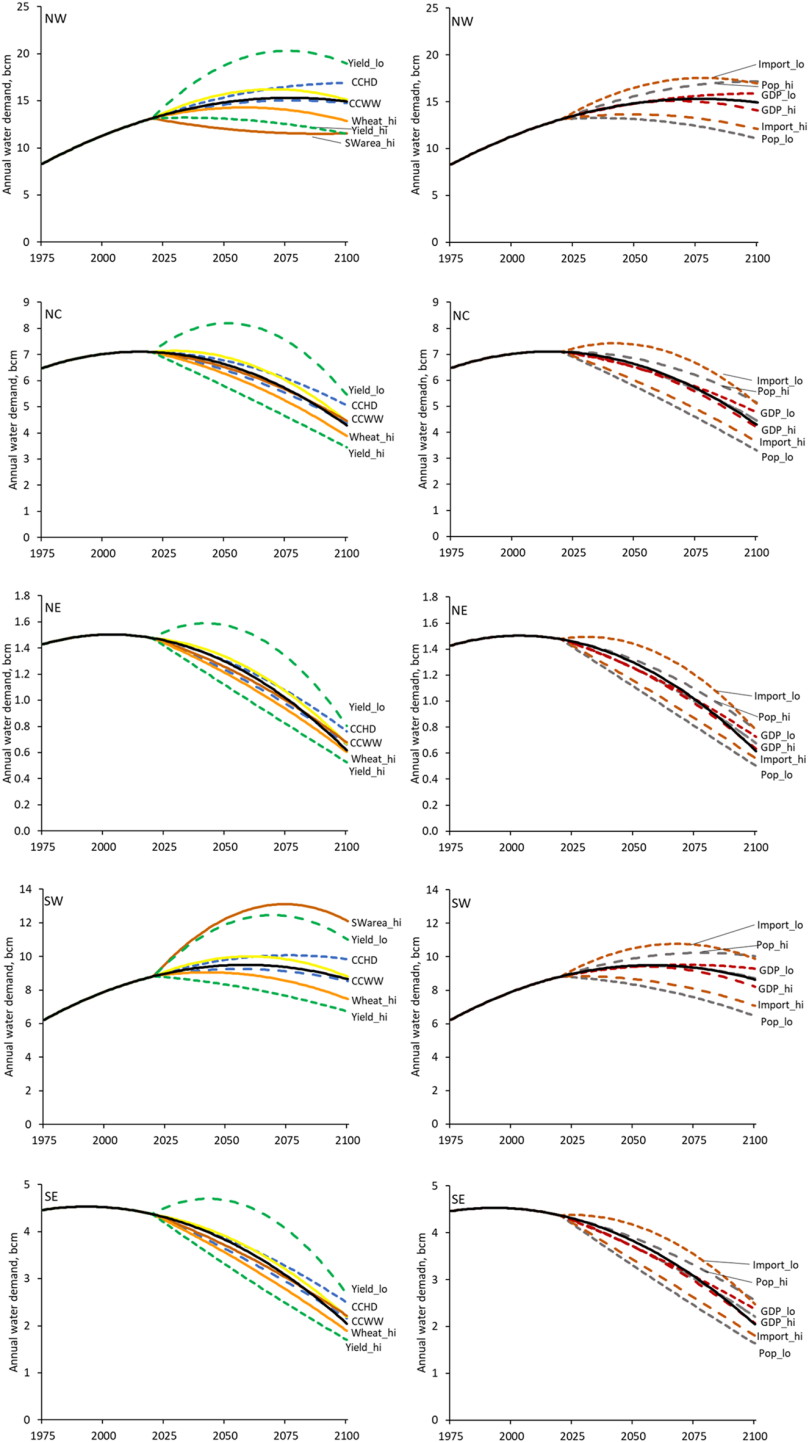
Volume of irrigation water demand in the scenarios for the five regions of Bangladesh. All curves are fitted polynomials, as shown in Fig. 4. The thick black line shows the base case in each plot. Note that the scale varies from region to region.

Figure 5 shows the volume of irrigation water demand in all scenarios, for each of the five regions of Bangladesh. The left column of plots shows the irrigation water demand for scenarios that assume mainly physical changes to cropping or, in the case of the climate change scenarios, to water supply and demand. The right column of plots shows scenarios that are primarily socio-economic; changes to population, GDP, and food imports. The labels for the lines on the two rows of charts indicate the scenario. Not all scenarios are listed, however, with those not listed being indistinguishable from the base case at the scale of the plot; they are the two rural to urban migration scenarios and the increased maize cultivation scenario. The increased area of cropping in the southwest region affects only that region and the northwest region. The figure shows that there is greater future demand for irrigation water than in the base case in the low yield increase, hot-dry climate change, low food import, high population growth and low GDP growth scenarios. Conversely, there is less future demand for water in the high yield increase, increased wheat production (due to a change in preference for wheat), low population growth, high food import and high GDP growth scenarios. The warm-wet climate change is little different from the base case, as are the two rural to urban migration scenarios and the increased maize cultivation scenario (noted above as not plotted in Fig. 5). The development of agriculture in the southwest leads to an increase in demand there and a corresponding decrease in demand in the northwest.

**Figure 6:**
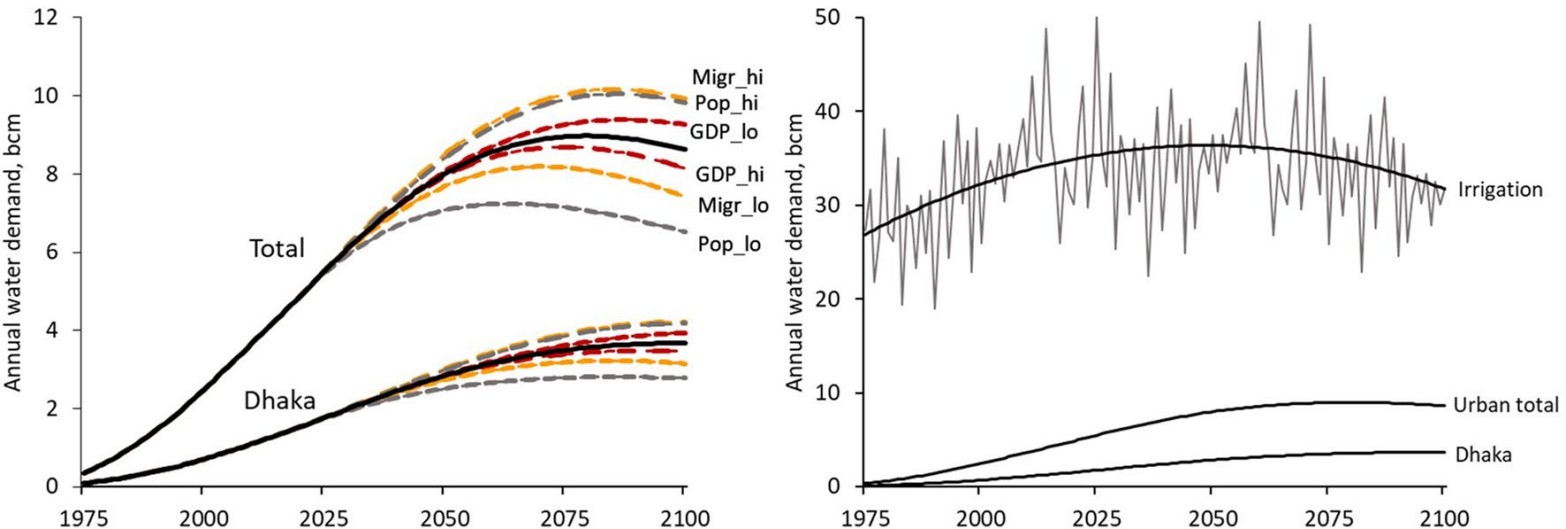
Volume of water demand for urban areas, total and Dhaka only, under a range of scenarios (left hand plot). The black lines are the base case (business as usual). The scenarios (dotted, colour lines) are labelled in the case of the total urban demand; the Dhaka scenarios have the same line colour and format, and occur in the same position relative to the base case. The volume of water demand in the base case for urban (total and Dhaka only) is compared with the base case Bangladesh total irrigation demand (right hand plot). For irrigation, the projected actual demand is shown as the thin grey line, with the fitted polynomial shown as the black curve.

The projected urban water demand in the scenarios is shown in Fig. 6. The figure shows the total urban demand, and that of Dhaka only. The demand for water rises above the base case for scenarios with a higher urban population (with higher national population growth, lower GDP growth, or a higher rural to urban migration rate); conversely, the water demand is lower for scenarios with a lower urban population. For comparison, Fig. 6 also shows the urban water demand and the total irrigation water demand (all five regions combined) for the base case. The urban water demand grows relative to the irrigation water demand, and by 2100 it is about one quarter of the national irrigation water demand.

## Discussion

In the base case, many features of the simulation (including population, GDP, food production, crop yield, areas of crops, and demand for water) compare reasonably well to the historical values for the period 1975–2020^34^.

The results show that the volume of irrigation water demand in Bangladesh is likely to increase in a business-as-usual scenario, due to population increase and increased consumption, offset somewhat by increased crop yields. The increase is concentrated mainly in the northwest, which has experienced greater increases in crop area in recent decades than have other regions. In the northwest, the increasing demand could exacerbate falling groundwater levels. The southwest is also projected to experience increasing irrigation water demand, though this is projected to peak there around 2060, and thereafter decline slightly. In a business-as-usual scenario, the irrigation water demand in other regions is not projected to increase much beyond present levels, and indeed could soon start to decrease.

Several factors could raise or lower the projected irrigation water demand from the business-as-usual case. The demand projections are affected more by the crop yield increase assumptions than by other assumptions used here. Most studies of yield increases in Bangladesh, including those mentioned in what follows, refer to rice and/or wheat only. Rice and wheat yields have increased substantially in recent decades^15,17,47–49^. Yields in current rice farming systems are about 50% below the potential yield^50^, and 20 to 30% below those that can be achieved with better practice^51–58^. Continuing large increases in rice yield are regarded as achievable^59^. With improved varieties and practices in the future, the assumed yield increases therefore appear plausible. On the other hand, future rice and wheat yield increases may be limited (or even reversed) by climate change^47,60^. However, the impacts of climate change on yield may be offset by practices such as changed rice transplanting dates^61,64^. To account for these various effects, our scenarios include a scenario with limited growth in yields above current yields, a middle (business-as-usual) and a high yield growth scenario.

Faster or slower population growth than that projected in the UN Population Division median projection has the next greatest impact on demand projections, after the crop yield assumptions. As noted in the methods section, the assumed population growth scenarios are well within the UN Population Division’s + /− 80% probabilistic scenarios (UN, 2019b), and are therefore not extreme assumptions. While we have treated population growth and climate change as independent at the country level, population growth and climate change are linked at the global level^65^, inasmuch as different socio-economic pathways (which include widely differing population growth trajectories) lead to different climate change outcomes.

Bangladesh in recent decades has approached self-sufficiency in food production, but nevertheless imports about 10–15% of its rice, wheat and maize^19,43^. While some authors discuss policies for achieving food self-sufficiency^19,50^, Bangladesh does not have a comparative advantage in growing wheat, and should import wheat while focussing production on rice, maize and potatoes^66^. Our scenarios of changing the fractions of crop requirements that are imported covers the possibilities of both greater and lesser self-sufficiency in the future. The impact on demand projections of changing the fraction of food imported in our scenarios is similar to that of the changed population growth scenarios. As noted in the methods section, our model does not simulate the economics of imports and exports, but treats them simply as a deposit into, or withdrawal from, an external pool. We think that this limitation is appropriate for our analysis.

Bangladesh is considered to be particularly vulnerable to climate related disasters and to climate change^60,67^. However, our results suggest that the direct effects of most extreme hot-dry projection in the most extreme IPCC SSP5-8.5 scenario results in a smaller impact on water demand for irrigation than the scenarios noted above. A wetter scenario barely differs from the projected demand without climate change. This is not to say that climate change is unimportant; indirect effects will likely include changes to crop yields which, as shown above, do have a large impact on the results.

Food preferences shifted from 1961 to 2013 to double the proportion of wheat in the diet^68^. A continuation of this trend, with a corresponding lowering of rice consumption, would decrease irrigation water demand. Increasing the proportion of wheat in a rice-based diet leads to better health and also lowers greenhouse gas emissions^69^. This factor in our scenarios has a similar magnitude of impact to that of an extreme hot-dry climate change, but in the opposite direction. On the other hand, a scenario with an increase in maize cropping for meat production had only a minor effect on the volume of irrigation water demand.

It may be noted from the above that several factors that are (in principle) within Bangladesh’s control (such as imports, a shift to wheat, population growth, etc.) have a greater impact than the most extreme hot-dry climate change scenario. This implies that, although climate change is projected to impact Bangladesh greatly, it is likely to be within Bangladesh’s capacity to manage the impact on water demand for irrigation. Although we have not modelled the scenarios in combination, the impact of factors within Bangladesh’s control would be greater if factors acted together (as shown in a study of water supply and groundwater demand in Pakistan^35^), reinforcing the implication that Bangladesh is likely to be able to manage the impact of climate change on water demand.

Of particular interest for the sustainable use of groundwater in the northwest, concentrating future crop area development in the southwest could decrease demand in the northwest more than most other factors. Increased cropping in the southwest is government policy^27^. Management systems have been developed that very significantly increase crop yields and production in the coastal zone of the southwest^51–54,57,58,63,70–74^. Hence northwest groundwater problems could be eased greatly with right policy mix, even in the face of extreme hot/dry climate change.

Across Bangladesh as a whole, the area available for cropping is declining at about 1% per year as arable land is converted to other uses^29,32^. Thus, the area of land available for cropping may limit crop production in the future^15,50,75^. Our results suggest that the areas of cropping required to satisfy future food requirements (under the assumed yield increases and levels of food imports) are, in much of Bangladesh, no greater than the areas currently under crops. This results partly from an increase in yields, and partly from an increase in the average number of crops grown each year on a piece of land, projected from the historical trends. Despite problems with salinity and limited water supply for irrigation, with appropriate management there is also great potential to expand cropping in the coastal zone^57,58,70–72,76,77^ and elsewhere^78^, including under conditions of climate change^70^ and sea level rise potentially leading to greater flooding^77^. In addition, there are trials with increased cropping intensity of four crops per year^79^, while agricultural land could be used more optimally by increasing the area under pulses, oilseeds and spices, and to limit the area under potatoes^18^. Whether these factors are enough to offset the declining area of arable land will depend on the impacts of Bangladesh government policies to limit the conversion of agricultural land to other uses. Currently, there is no policy to protect agricultural lands for conversion to urban and industrial use. However, there are increasing concerns at the policy level about the protection of agricultural lands to sustain crop production and food security, and a bill has been tabled in the Bangladesh Parliament for the proper use and protection of agricultural land (https://bdnews24.com/economy/2022/03/31/bill-in-parliament-to-protect-agricultural-land).

While irrigation is the greatest user of water, particularly groundwater, groundwater pumping for urban water supply (generally from deeper parts of aquifers than the shallow pumping for irrigation) is nevertheless a concern. The use of groundwater to supply Dhaka already causes concerns over regional impacts of contaminant migration and potential contamination of deep aquifers^25^. Groundwater depletion may also be a problem in other large cities such as Chittagong^80^. Urban water demand is projected to increase to approximately double the current level. The projected increase is impacted by overall population growth (and hence also GDP impacts on population growth) and by rural–urban migration. The latter impact could see urban water demand continue to grow after the overall population of Bangladesh has ceased to increase. The projected growth in urban water demand is a greater proportionate increase than that of irrigation water demand. If groundwater continues to be a major source of water for large urban centres, it therefore seems likely that the concerns over the unsustainability of use will grow more acute in the urban case than in the irrigation supply case.

## Conclusions

We conclude that, consistent with the findings of many studies in the literature, pressure on water use in Bangladesh is likely to increase in coming decades. In particular, irrigation water demand in a business-as-usual scenario in the northwest is likely to increase for much of the coming century, potentially exacerbating concerns about sustainable use of groundwater. However, several policy options could reduce this pressure in the northwest, including policies that shift future growth in irrigated cropping to the southwest, shift crops away from rice (particularly Boro rice) perhaps due to a change in food preferences, and limit population growth (perhaps in concert with increased GDP growth).

Urban water demand is also projected to grow, to about double the current level. In the case of Dhaka in particular, this would place great pressure on groundwater supplies unless alternative sources are developed.

## Data Availability

The datasets used and/or analysed during the current study available from the corresponding author on reasonable request.
